# Knowledge Is Power*Address delivered before the North Texas Medical Association at Bonham, Texas, June, 1899.

**Published:** 1899-08

**Authors:** John O. Scott

**Affiliations:** Sherman, Texas


For the Texas Medical Journal.

### Knowledge is Power.*

BY JOHN O. SCOTT, M. D., SHERMAN, TEXAS.

The man of the healing art, from the renowned surgeon, who enjoys palatial surroundings, to the most obscure country physician, is the people's friend.

His heart is ever open to the calls of humanity. There is always a tender chord of sympathy in his breast for the suffering. His love for the human race is universal. "Don't cheer, boys; the poor devils are dying," the merciful words of Captain Philip, express in becoming language the love which is ever in the doctor's heart for the human kind, be they friend or foe; be they the wild man of the forest or the honored peers of civilization. " 'Tis only noule to be good; kind hearts are more than coronets."

When the weeping Breisis, radiant with ravishing beauty and adorned with utmost luxuriance of personal charms, viewed "on the cold earth divine Patroclus spread, pale and gashed with cruel wounds," she mournfully uttered this most tender sentiment of sympathy: "Accept these grateful tears, for thee that ever felt for another's woes." In dulcet symphonies of rapturous verse Homer exalts this lovely woman with the noblest sentiment ever composed by those who attune their harps to measured lays or harmonious rhythms. It was that holy fervor burning in their breasts "to ever feel another's woes" that during the yellow fever epidemic impelled Drs. Moody, Hank, Covey, Hayes, Gant, Alexander, Manning and other Texas physicians like heaven-born heroes to meet death face to face and from his gory hands to receive the chaplet of immortality. It was that sublime passion "to ever feel for another's woes" that induced Clara Barton to embark for the West Indies and with her own hands give a healing balsam to the suffer-

---

*Address delivered before the North Texas Medical Association at Bonham, Texas, June, 1899.

ing soldier. It was that noble sentiment to "even feel for another's woes" that at the battle of Murfreesboro influenced Dr. Legre to go into the very jaws of death in the dreadful storm of the raging conflict and staunch the bleeding artery of the heroic Hanson.

Alexander is reckoned as one of the most renowned captains who ever figured in the world's history. He is known as a conqueror of many nations, but we remember him only in tragic events, of the happiness of others destroyed by his cruel sword, of kind Darius on bended knee begging for life, for mother, for daughter, for wife. Of his countryman, Hippocrates, the picture is different. We see a wise and knowing man, the benefactor of his race. His name is illumed with a bright halo of grandeur, the effulgence of his glory not stained with the blood of the innocent or bleared with the barbaric pearl or Pactolian gold.

The diadem of fame that crowns the brow of Hippocrates has been shining brightly through the rolling centuries with undimmed lustre, decked with priceless gems that never fade, the fruit that his genius wrought when inspired with the sublime sentiment, "knowledge is power, wisdom a crown of glory."

The name of Julius Cæsar is transmitted through history as a skillful general, to marshal armies in the "crimson paths of war." Across the gulf of centuries the blood of his tragic death still stains the Roman forum. The gory dagger of Brutus is yet the nightmare to crowned tyrants.

The name of Galen, through the mysterious darkness of the middle ages, has been a brilliant luminary in science—an ornament to the Roman State, the brightest gem on the brow of time.

He is esteemed a benefactor to his race, giving to medicine its theory, accomplishing much to deserve the gratitude of mankind and place his name in the galaxy of eternal fame. Divinely blessed with knowledge and wisdom he has been honored by posterity with a crown of glory.

When the Duke of Marlborough, seizing the golden opportunity for distinction, marched his veteran legions to Prince Engene's relief at Blenheim, like a hurricane blast he struck the advancing foe and wiped them out of existence. The ringing of bells and salvos of artillery resounded on the hill-tops and through green valleys, all over the United Kingdom, in honor of this wonderful achievement of British prowess. The artistic genius of the painter's brush was sought to picture on canvas the victories of Marlborough to adorn the public halls of the nation. The Parian marble, wrought into statuary by the most gifted sculptor, stood in proud eminence in the parks of the metropolis in memory of that illustrious general.

History records another Englishman whose fame is not confined to the realms of that empire, "upon whose flag the sun never sets." Instead of one nation, the entire civilized globe eulogizes the name of Jenner. The people of the wide, wide world speak of him as a benefactor of his race. His fame was not made universal by the bloody sword of the conqueror, like Marlborough, nor do the people remember him in enchanting verse or inspiring lays like Milton, or the bard of Waverly; nor is he remembered by words of wisdom scattered in tragedic verse, like brilliant diamonds, as Shakespeare, nor is his name transmitted, illustrious, to posterity in utterances of burning eloquence, as Grattan or Burke. In every hamlet, in every hospital and in every home, all over the globe, there can be heard the honored name of Jenner. The story of the milkmaid, and Jenner's discovering the immunity from small-pox, is known in every land. In palace or cottage, whenever there are found parents or loving children, his memory will be held in tender gratitude. Illustrious Jenner! The grandeur of his deeds, the munificence of his offerings to suffering humanity, are not like the glittering diamond shining in princely diadem, but as a crystal fountain, flowing gently from the mountain side, giving cooling drink to thirsting humanity. Renowned Jenner! He attained the acme of his fame by industry, by observation, by study. He reached his eminence by the power of his knowledge. He gained his crown of glory by his wisdom.

When Wellington, with his invincible heroes at Waterloo, stood immovable as the Grecian phalanx, opposing the desperate charges of the old guard, the entire British empire lauded him to the skies. The shouts of praise could be heard from the hetherclad hills of Scotland to the sea-roaring coast of Cornwall.

At Falkstone there is an unpretentious marble urn to the memory of Harvey. Beneath his name is chiseled, "Discoverer of the Circulation of the Blood." As the flowers of Autumn, before December's chilly blasts, fade from the face of the earth, so, by the destroying hand of time, the name of Wellington and of Waterloo will be effaced from the page of history. So long as time endures will Harvey's name be cherished as a benefactor to his race. Harvey performed his deeds of eminence by long years of study, toil, temperance and industry. The laurel wreath that graces his brow dazzzles with the words of inspiration, "Knowledge is power, wisdom a crown of glory."

All over England there are monuments and statues in commemoration of Nelson's victory. On the glistening surface of the polished marble is inscribed his battle signal, "England expects every man to do his duty." When the blood-crimsoned word "Trafalgar" has faded from the memory of man; when the poet ceases to sing in raptured lays the chivalric deeds of that battle; when the orator, in thrilling eloquence, no longer tells to listening multitudes that wondrous naval victory, the poor, wounded soldier will yet be grateful to Lister, that by his genius his life has been saved.

When the marble columns to Nelson's memory have perished, in every niche, in all the hospitals of the civilized world there will be statues to the memory of the man who first conceived aseptic surgery. Millions of grateful beings now sing peans of praise to their living benefactor. The learned of earth have painted on the escutcheon of fame his illustrious name in exalted eulogy. On all the proud monuments that posterity will erect to his memory, in bold relief, in shining character, will be inscribed, on the topmost pediment, the golden motto of the people's friend, "Knowledge is power, wisdom a crown of glory."

Napoleon's name, like a blazing comet, is still flaming across the skies—the admiration of millions, the world's amaze. In the Louvre the walls are decorated with costly paintings of his victories. The genius of David and Vernet have given to the people in lifelike colors pictures of Austerlitz, Marengo and Friedland.

Bonaparte is regarded the most gifted military genius of this century. His wonderful victories are yet the theme of the historian —to dip his bloody pen and write of heroes and renown.

We blend his name with the weeping Josephine, the gory wounds of the murdered St. Enghein—the thousands who perished on the frozen steppes of Russia. The laconic words of Baron Larrey under the shadows of pyramids, "My profession is to cure, not to kill," are to the fame of Napoleon like "golden apples in a silver picture."

In the cemetery at Lexington, Ky., there can be seen from afar a grand masterpiece of the sculptor's skill. From a score of roads converging to the city the traveler views this lofty cenotaph,grandly rearing its massive column to the skies. Upon the crowning capstone of that vast architectural design is placed the statue of Henry Clay, with lips that almost speak carved from the Italian marble. Today not a schoolboy can narrate a deed that Clay has rendered for the good of his country—some benefit to his fellows in distress.

In a private burial ground in Danville, Ky., we doubt if one can find the grave of Ephriam McDowell. There is not an intelligent woman in all America but can tell the unspeakable good McDowell has rendered female kind. His name will shine throught the ages on the highest pinnacle of fame, bright as the brilliant light on lofty tower to benighted mariners midst angry waves or frowning rocks. His inventive genius, his boldness in operation, his knowledge, his wisdom, won the palm of victory, the crown of glory, fadeless as the starry diadem on the brow of night.

At Buena Vista General Zach Taylor fought a memorable battle, gaining a signal victory, which has been sung in poetry and written in prose.

After many years of labor, self-denial and study, Long discovered anæsthesia, and was the first to perform a painless operation under its benign influence. The honored name of Long grows brighter as each revolving year unloads into the lap of recording fate its collection of events.

When that lofty monument at Frankfort which, in imperial grandeur, casts its colossal shadow across the pearly stream that bathes its base, and tells the green valleys and the giant hills of Taylor's victory—when its huge polished stones and marble statues, like the works of art of Ninevah, are buried in the sand, mankind in grateful memory will cherish the name of Crawford Long. Dr. Long, like the discoverer of America and the inventor of the cotton gin, died broken hearted, that his labors were not recognized by his contemporaries. A grateful posterity, for his knowledge will give him his reward; for his wisdom he wears a crown of glory.

In a country town in South Carolina Marion Sims began his medical career. For long years he endured the bitter pangs of penury. After many months of labor Sims devised the silver wire suture and made a speculum which now bears his name. Assisted by these instruments he successfully operated for vesico-vaginal fistula and other female infirmities, which has placed his name among the illustrious men of the world's history—a shining light among the luminaries of fame. When he visited Europe in 1865 princes and paupers alike shared the working of his beneficent genius. His arrival in cities was heralded as one blessed with superhuman power to heal the afflicted. During that same eventful period there were many who lauded the military genius of Stonewall Jackson. His name was pronounced in eulogy by potentate and beggar. The fame of Jackson shone across the heavens like the dazzling light of the meteor beam. The halo of his fame was crimsoned with the sacred blood of thousands of dead heroes.

The name of Sims, like a fixed constellation in the starry firmament, shines with a steady glow of brilliant light. To thousands of human beings it was the star of freedom from agonizing pain. To millions it has been the brightest morning star—the star of joy for renewed health.

In New York City the doctors of the world have erected a memorial to his fame, because of his eminent achievements. His learning, his industry, his knowledge gave him power; his heaven-born wisdom a crown of glory.

One by one the majestic oaks of the forest fall beneath the woodman's ax. In time there is neither twig nor leaflet remaining to declare their former grandeur and gorgeous beauty. During the going and coming of centuries the renowned deeds of warriors and statesmen have faded from the memory of man, and there is nothing to remind posterity of the good they may have performed.

The achievements of the medical man, great and small, through the circling years, since creation's dawn, make a monumental pile, reaching to the very courts of heaven. Sweet charity, adorned with the utmost luxuriance of celestial beauty, graces the topmost summit. Upon her snow white breast she wears the jeweled cross of love; around her peerless waist is clasped the magic girdle of woman's charms—charms to soothe the troubled heart; charms to soothe the sick man's sufferings; charms to soothe the dying agony. Her noble brow shines with a radiant crown. It is the crown of knowledge; it is the crown of wisdom; it is the crown of eternal glory.

